# Comparison of Phenolic Profile of Balsamic Vinegars Determined Using Liquid and Gas Chromatography Coupled with Mass Spectrometry

**DOI:** 10.3390/molecules27041356

**Published:** 2022-02-17

**Authors:** Michal Kašpar, Tomáš Bajer, Petra Bajerová, Petr Česla

**Affiliations:** Department of Analytical Chemistry, Faculty of Chemical Technology, University of Pardubice, Studentská 573, CZ-532 10 Pardubice, Czech Republic; michal.kaspar3@student.upce.cz (M.K.); tomas.bajer@upce.cz (T.B.); petra.bajerova@upce.cz (P.B.)

**Keywords:** balsamic vinegar, phenolic compounds, antioxidants, liquid chromatography, gas chromatography

## Abstract

Balsamic vinegar is one of the best known and most popular types of vinegar, and it is a rich source of polyphenolic compounds. The quality of balsamic vinegar as well as the content of phenolic substances vary depending on the production method. In the present work, we have developed a method for comprehensive characterization of the content of phenolic compounds in balsamic vinegars based on the combination of gas chromatography (GC) and high-performance liquid chromatography (HPLC) coupled with mass spectrometric detection in single mode (MS) and tandem mode (MS/MS). In total, 14 samples of different types of balsamic vinegar were analyzed without difficulty in sample preparation. The separation conditions and detection parameters of HPLC-MS/MS were optimized and used for the determination of 29 phenolic compounds and 6 phenolic acids. The profile of phenolic compounds was completed by semi-quantitative analysis of volatile organic compounds using GC-MS after optimized headspace solid-phase microextraction. Gallic acid, protocatechuic acid, caffeic acid, and *p*-coumaric acid have been identified as the major phenolic compounds in balsamic vinegars.

## 1. Introduction

The term “phenolic compound” is used for aromatic organic substances containing one or more hydroxyl groups directly bonded to a benzene ring. Typical natural sources of these compounds are plants, from which they are further processed into plant products [1]. Phenolic compounds significantly affect the color, taste, and aroma of products [2], and have a positive effect on the human body [3]. Reactive oxygen species such as superoxide, hydrogen peroxide, and hydroxyl radicals affect lipids, membranes, proteins, and DNA in the human body. This can result in accelerated ageing, cancer formation, and brain degeneration [4]. Phenolic compounds have high antioxidant activity, which can help the human body prevent oxidative stress [5]. Phenolic compounds have been shown to help the body fight cancer [6] and cardiovascular diseases [7].

Balsamic vinegar is a rich source of phenolic compounds [8]. The tradition and history of its production are closely linked to the Italian provinces of Modena and Reggio Emilia. Therefore, in 2000, it obtained a protected designation of origin, which allows products called “traditional balsamic vinegar of Modena” (TBVM) or “of Reggio Emilia” (TBVRE) and “balsamic vinegars of Modena” (BVM) or “of Reggio Emilia” (BVRE) to be produced only in these Italian provinces and by using specified methods. Other products called “Balsamic vinegar” can be produced anywhere in the rest of the world [9]. The production of traditional balsamic vinegars begins with the concentration of grape must on an open fire. This is followed by a first fermentation (conversion of sugars to ethanol by yeast) and a second fermentation (conversion of ethanol to acetic acid by acetic acid bacteria). Subsequently, the vinegar is aged in a series of five wooden barrels (each of a different type of wood) for at least 12 years. During this time, the barrels are refilled to compensate for liquid losses during evaporation. No additives may be added to the vinegar and no processes may be carried out to accelerate the ageing of the vinegar [10,11,12]. The production of BVM and BVRE is much simpler and, above all, several times faster. It is based on mixing of cooked wine must with wine vinegar, and additives (e.g., caramel) may be used during production [13].

There are several studies focused on the analysis of phenolic compounds in balsamic vinegars using reversed-phase (RP) liquid chromatography. Liu et al. [14] used the HPLC-DAD method to detect phenolic acids in 23 samples of vinegar, including balsamic vinegar. A Zorbax Extend-C18 column and gradient elution (methanol-formic acid 0.1%) were used for separation. The HPLC-DAD method was used by Bakir et al. [15] for the analysis of phenolic compounds in vinegars. Separation was performed using a Luna C18 column in gradient elution mode with aqueous/acetonitrile mobile phase containing 0.1% trifluoroacetic acid. Natera et al. [16] carried out the analysis of phenolic compounds using the HPLC-DAD method with a LiChrospher 100 RP-18 column, and the mobile phases used were 5% methanol with 95% water–95% methanol with 5% water at pH 2.5. Cerezo et al. [17] performed phenolic compounds analysis using the HPLC method coupled with a UV/VIS detector. The separation column used was a LiChroCART 250-4 Superspher 100 RP-18. Gradient elution was utilized using acetonitrile/water with the addition of acetic acid yielding pH 2.65. All these methods utilized columns packed with 5 μm particles and relatively long analysis times (45–75 min). Barnaba et al. [8] conducted an extensive study of phenolic compounds. The HPLC-MS/MS method with online solid phase extraction (SPE) was used for the analysis of 56 phenolic compounds in various spirits, wines, and vinegars. Several types of SPE cartridges were tested, and the HyperSep^TM^ Retain PEP cartridge packed with porous polystyrene-divinylbenzene material modified with urea functional groups showing the most balanced retention for the tested compounds. The Acquity UPLC BEH C18 column was found to be the best for the separation of phenolic compounds. A gradient of acetonitrile–water was used for elution.

In recent years, comprehensive two-dimensional liquid chromatography has been used for the analysis of phenolic compounds in food samples [18,19]. The most commonly used phase systems are RP × RP followed by a combination of RP with hydrophilic interaction liquid chromatography. Although the two-dimensional LC method has not yet been reported for the analysis of polyphenolic compounds in balsamic vinegar samples, it is widely applied for the analysis of polyphenols in grapes and other products of grapes [20,21], for example, wine samples [22,23].

The analysis of volatile compounds presented in balsamic vinegar samples is usually performed using gas chromatography coupled with mass spectrometry (GC-MS). Thus, Plessi et al. [24] used the GC-MS method for the analysis of phenolic acids after derivatization by *N*,*O*-bis(trimethylsilyl)trifluoroacetamide (BSTFA). Different SPE cartridges were tested (Chem-Elut cartridge and polyamidic cartridge). Separation was performed using an RTX-5MS capillary column. Sinanoglou et al. [25] used a similar method, but with SPE on the C18 isolate column. Both methods use a derivatization step prior to the analysis, which can increase the volatility of the compounds, but can be time-consuming and risk contamination or degradation of the sample.

In this study, our aim was to develop a method for the comprehensive characterization of phenolic compounds in balsamic vinegars based on a combination of HPLC coupled to single- and tandem mass spectrometry (HPLC-MS, HPLC-MS/MS) and GC-MS. We have optimized the extraction conditions of sample preparation for GC-MS analysis using the design of the experiment, and we optimized the separation and detection conditions for the HPLC method. We have applied the developed method to 14 samples of balsamic vinegars of different qualities and compared the composition of the samples with respect to the volatile compounds determined using GC-MS and the phenolic compounds determined using HPLC-MS(/MS).

## 2. Results

### 2.1. Selection and Optimization of Extraction Techniques for GC Analysis

In the initial experiments, two extraction methods were compared, i.e., static headspace (HS) extraction and solid-phase microextraction from the headspace (HS-SPME). The results (see Supplementary Materials Tables S1 and S2) indicated that both compared parameters, that is, the number of peaks and the total peak area of the compounds presented in the chromatograms, were significantly higher for the HS-SPME method compared to the HS extraction method. Therefore, the HS-SPME method was chosen for further analyses.

When comparing the HS-SPME results for different fibers (Table S2), it was found that slightly more compounds were extracted using PDMS (polydimethylsiloxane) fiber; however, peak areas were many times higher when extracted on DVB/CAR/PDMS (divinylbenzene/carboxen/polydimethylsiloxane) fiber. The lower total peak area achieved for PDMS fiber preferably corresponds to the extraction of nonpolar compounds, which is also indicated by the higher peak area achieved for a sample diluted with saturated NaCl solution by the salting-out effect on the polar compounds. The higher total peak area obtained for DVB/CAR/PDMS fiber can be attributed to the bipolar characteristics of the fiber, yielding both absorption and adsorption affinity of the fiber for both low-molecular-weight volatile compounds and the polar compounds presented in the vinegar sample. Therefore, the HS-SPME extraction on DVB/CAR/PDMS fiber was chosen for further optimization of the sampling process. 

**Figure 1:**
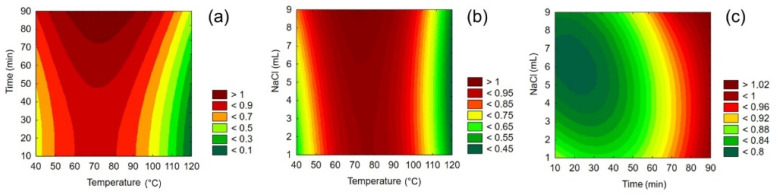
Suitability of the extraction conditions as a function of (**a**) extraction temperature and extraction time, (**b**) extraction temperature and volume of NaCl solution, and (**c**) extraction time and volume of NaCl solution determined by the response surface method evaluation of central composite design of extraction.

The results of the HS-SPME optimization are summarized in Table S3. The evaluation of the central composite design was based on the number of peaks in the chromatograms. Peaks with a signal-to-noise ratio less than three were excluded from the evaluation. Based on the response surface analysis (see Figure 1), the optimal extraction temperature was 75 °C. Figure 1 shows that the number of peaks in the chromatograms increases with increasing extraction time and the amount of saturated solution. An extraction time of 90 min was considered sufficient. The amount of saturated NaCl solution is limited by the volume of HS vials used, 9 mL being the maximum. Using the polynomial Equation (1), it was calculated that using 6 mL of NaCl solution would theoretically yield a similar number of peaks as using 9 mL of NaCl solution (139 versus 142 peaks). Thus, the volume of 6 mL of saturated NaCl solution was selected, which increases the vapor space in the HS vials used.

(1) $$\begin{array}{cc} NoP=3.257+ & 2.762\times X-0.017\times X^{2}-0.592\times Y+0.007\times Y^{2}+4.414\\ & \times Z-0.266\times Z^{2}+0.0004\times (X\times Y)-0.035\times (X\times Z)\\ & +0.035\times (Y\times Z) \end{array}$$

*NoP*—number of peaks; *X*—extraction temperature; *Y*—extraction time; *Z*—volume of saturated NaCl solution.

**Figure 2:**
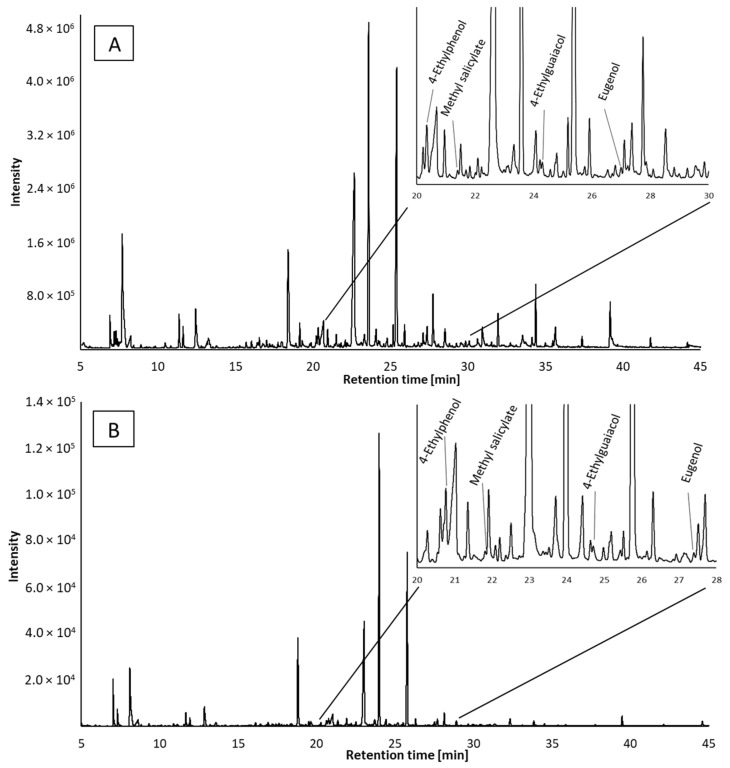
Gas chromatographic analysis of volatile organic compounds in sample 14 ((**A**)—MS detection; (**B**)—FID detection).

Optimized extraction conditions were used for semi-quantitative analysis of samples of balsamic vinegars using gas chromatography with flame-ionization (GC-FID) and mass spectrometric detection (GC-MS). Representation of typical chromatograms of volatile organic compounds (VOCs) in the balsamic vinegar is shown in Figure 2A for GC-MS and Figure 2B for GC-FID methods. The magnified segment of the chromatograms contains compounds that have been quantified, and the results of the semi-quantitative analysis are summarized in Table S4. The compounds were assigned on the basis of the comparison of MS spectra and retention indices.

### 2.2. Optimization of HPLC-MS/MS Conditions

**Figure 3:**
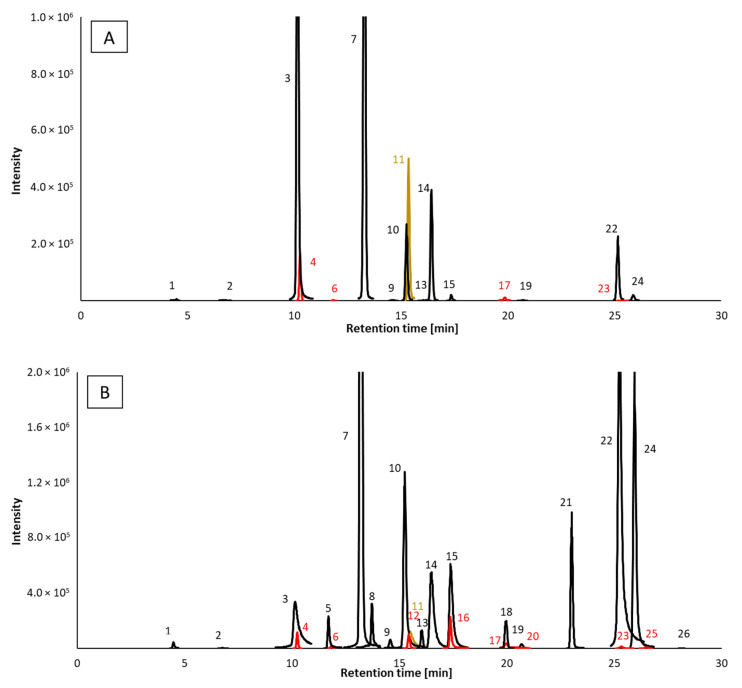
HPLC-MS/MS separations of phenolic compounds ((**A**)—sample No. 14; (**B**)—standard mixture) 1—Pyrogallol; 2—Eugenol; 3—Protocatechuic aldehyde; 4—Tyrosol; 5—Catechin; 6—Homovanillyl alcohol; 7—4-Hydroxybenzaldehyde; 8—Epicatechin; 9—4-Methylcatechol; 10—Vanillin; 11—Ethyl gallate; 12—Coniferyl alcohol; 13—4-Hydroxy-3-methoxyphenylacetone; 14—Syringaldehyde; 15—Scopoletin; 16—Rutin; 17—4-Hydroxy-3-methoxycinnamaldehyde; 18—Tryptophol; 19—2,6-Dimethoxyphenol; 20—Salicylaldehyde; 21—Resveratrol; 22—Ethyl-3,4-dihydroxycinnamate; 23—4-Vinylphenol; 24—Ethyl vanillate; 25—2-Methoxy-4-vinylphenol; 26—4-Ethylguaiacol. Concentration of standards 1mg/L each; chromatograms with different colors correspond to the compounds that coelute but are distinguished by different MRM transitions, as shown in Table S5.

Based on the results of our previous studies with the separation of phenolic compounds [26], we have selected reversed phase separation conditions. We have tested two columns packed with stationary phase of the octadecyl silica gel, that is, the Luna C18 and Luna Omega PS C18 phase (both 150 × 3.0 mm, particle size 3 μm particle size). The difference between the columns is in the mixed-mode stationary phase with embedded functional groups with positive charge for the PS C18 phase, improving the retention of acidic compounds. For the balsamic vinegar samples, we have, however, achieved better results in terms of peak shape using the Luna C18 column. We have tested different gradient profiles for the aqueous (A)/acetonitrile (B) binary mobile phase, among which the best separation was achieved using gradient profile as follows: 3–48.4% B from 0 to 35 min, 48.4–100% B from 35 to 35.1 min, 100% B from 35.1 to 38 min, 100–3% B from 38 to 38.1 min, 3% B from 38.1 to 40 min. Although several compounds coeluted even under optimized gradient conditions, we have used MS/MS detection in the multiple reaction monitoring mode (MRM). The conditions of MS/MS detection were optimized for the quantitative analysis of phenolic compounds. The obtained values (see Supplementary Materials, Table S5) of the declustering potential (DP), collision energy (CE), cell exit potential (CXP), and transitions were used for detection. Typical chromatograms of phenolic compounds obtained by the HPLC-MS/MS method are shown in Figure 3. The concentrations of the compounds detected and their standard deviations are presented in Table S6. The calibration curve parameters and detection limits are summarized in Table S7. 

## 3. Discussion

### 3.1. Gas Chromatography

**Figure 4:**
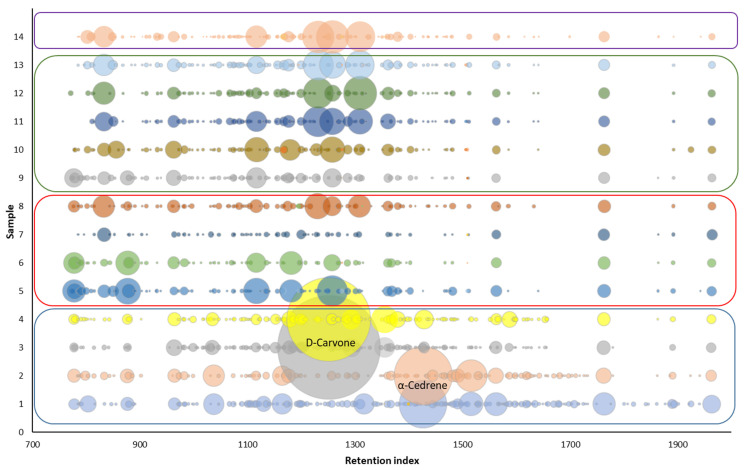
A comparison of the overall aroma profile of samples 1–14 obtained by GC-FID analysis. Blue box—Greek balsamic vinegar; red box—BVM; Green box—“premium“ BVM; purple box—TBVM. Spots represent each peaks of the compounds in chromatogram; the size corresponds to peak area of volatile compounds in chromatogram.

Plessi et al. [24] quantified nine phenolic acids by GC-MS after derivatization with BSTFA. In their work, gallic acid, protocatechuic acid, and *p*-coumaric acid were the main compounds with similar concentration levels (approx. 18 mg/L); however, the results of the quantitative analysis were not completely satisfactory due to the low recovery rates of extraction of phenolic acids. In our study, we performed GC-MS and GC-FID analysis without derivatization step; thus, we were not able to identify phenolic acids in the samples. Instead, we aimed at the overall profile of other phenolic volatile organic compounds. The presence of eugenol, 4-ethylphenol, 4-ethylguaiacol, 2,4-di-*tert*-butylphenol found in the samples corresponds to the published data [27,28,29,30,31]. In these articles [28,29,30,31], several other phenolic compounds were identified: syringol, guaiacol, 4-methylphenol, phenol, tyrosol, and 4-acetyl-2-methylphenol, which were not identified in our samples. On the other hand, we identified some compounds that were not previously reported in the balsamic vinegar samples: methyl salicylate, isopseudocumenol, thymol, 4-vinylguaiacol, 1-hydroxy-3,4,5-trimethylbenzene, allyl cresol isomer, vanillin, *trans*-isoeugenol and 2,6-di-*tert*-butylmethylphenol. The presence of thymol at a high content level (2.41%), and other compounds found only in sample No. 3 may be related to the addition of thyme honey to the vinegar [32], which the manufacturer declares (Table 1). Although the addition of thyme honey is not declared in sample No. 4, it is produced by the same manufacturer, and the results suggest that the certain amount of thyme honey can also be presented in this sample (3.05% of thymol). The addition of thymol can also be used as a flavoring agent [33]. A comparison of the overall aroma profile of individual samples is shown in Figure 4.

Samples No. 3 and No. 4 showed a significant *D*-carvone content compared to the other samples. This terpenoid is found in dill seeds, which are used to flavor, including vinegars [34]. Samples No. 1 and No. 2 contained α-Cedrene, the source of which is cedar wood oil. Therefore, its presence in vinegars may be related to the maturation of vinegar in barrels made of cedar or juniper wood [35]. Although the aroma of cedar wood oils is typically used in the production of soaps, room sprays, and disinfectants, it can also be used as a flavoring agent [36].

4-Ethylguaiacol along with methyl salicylate were identified in most of the analyzed samples (both compounds were found in nine samples of all types of vinegars). Methyl salicylate gives the vinegar a floral scent [37]. The aroma of 4-ethylguaiacol is characterized as spicy and is undesirable for the resulting aroma. The same applies to other ethylphenols, 4-ethylphenol with an aroma described as “animal”. Both of these ethylphenols have an origin in yeasts [38]. 4-Ethylphenol was identified only in three samples, but with a significant content of approximately 1%. 

### 3.2. Liquid Chromatography

**Figure 5:**
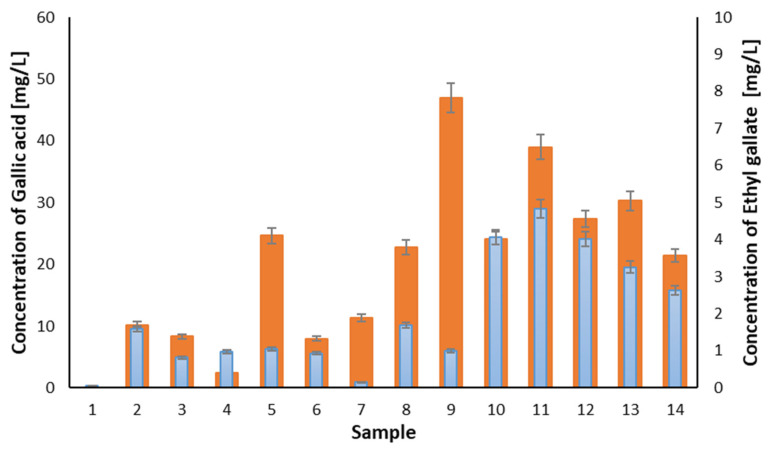
Comparison of the concentration levels of gallic acid (orange) and its ester ethyl gallate (blue) for set of samples of balsamic vinegars. Error bars represents standard deviation (*n* = 3).

The most abundant phenolic compound detected among all samples of balsamic vinegars was gallic acid (Figure 5), followed by other phenolic acids (i.e., caffeic acid, protocatechuic and *p*-coumaric acid; Table 2). The presence of these phenolic acids was also reported by Barnaba et al. [8], and this is in good agreement with our results. Other compounds commonly found in balsamic vinegars are protocatechuic aldehyde, ellagic acid, vanillin, and coniferyl alcohol (with mean concentrations 6.41, 3.76, 5.62, and 4.76 mg/L, respectively [8]). The mean concentration of protocatechuic aldehyde found in our samples was 1.42 mg/L and 0.476 mg/L for vanillin. Coniferyl alcohol was not identified in our samples, and the analysis of ellagic acid was not performed in this work.

Natera et al. [16] in their work also determined these four phenolic acids as the main compounds, but much higher mean values of the compounds were found in balsamic vinegars with respect to our samples. Furthermore, they quantified tyrosol as the compound with the highest mean concentration (i.e., 109.4 mg/L), contrary to Barnaba et al. [8], who reported a mean concentration of tyrosol of 0.273 mg/L. Tyrosol had a mean concentration of 1.697 mg/L in our study. Tyrosol is formed from tyrosine during the fermentation process and is directly proportional to the amino acid content in the grape must [39].

Liu et al. [14] determined gallic acid, protocatechuic acid, caffeic acid, and *p*-coumaric acid as the main compounds in BVM which agree with our results. Their concentration levels were similar to those of our work and the work by Barnaba et al. [8]. Plessi et al. [24] determined phenolic acids by the GC method and found the following concentrations in TBVM samples (*n* = 4): gallic acid (18 mg/L); protocatechuic acid (18.8 mg/L); caffeic acid (10.9 mg/L) and *p*-coumaric acid (17.1 mg/L).

Besides the phenolic acids, there were two compounds in the samples that were identified by both the HPLC and GC methods, i.e., eugenol and vanillin. Eugenol, which has a clove aroma, showed a higher content determined by HPLC in sample No. 5; however, semi-quantitative GC analysis indicated the highest content in samples No. 3 and No. 4. Vanillin was identified by GC only in samples No. 1 and 2 (the same trademark of the samples), which is consistent with the results of HPLC. Compared to the other balsamic vinegar samples, it is possible that vanillin was added artificially to samples No. 1 and No. 2 as a flavoring agent [40].

In our study, several other compounds (Table 2) with significant concentration levels were found. Salicylic acid, ethyl gallate, and protocatechuic aldehyde were determined in “premium” BVM and TBVM. Slight differences in the pyrogallol content were found between the types of balsamic vinegars. The variable content across the samples also showed tyrosol and syringic acid. 

**Figure 6:**
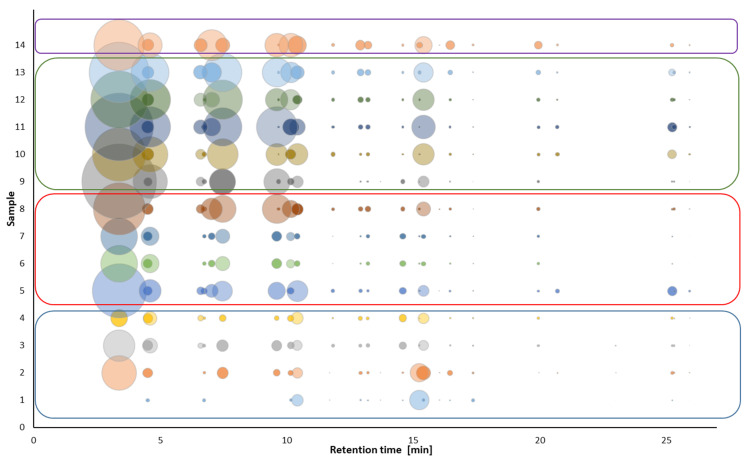
Comparison of the phenolic profile of samples 1–14 obtained by HPLC analysis. Blue box—Greek balsamic vinegar; red box—BVM; green box—“premium“ BVM; Purple box—TBVM.

There are significant differences in the composition of phenolic compounds, determined using the HPLC method, between the categories of balsamic vinegars (see Figure 6). The total phenolic content (mean ± standard deviation) for each category was found as follows: Greek balsamic vinegar (samples No. 2–4) (14.69 ± 5.01 mg/L); BVM (31.5 ± 17.6 mg/L); “premium” BVM (75.6 ± 13.4 mg/L) and TBVM (56.7 mg/L). From the results, it is obvious that the samples with the expected higher quality have a higher content of phenolic compounds. In sample No. 1 (white Greek balsamic vinegar), a significantly smaller total amount of phenolic compounds was detected.

There are also differences within the categories. In Greek balsamic vinegars, sample No. 1 was the only white balsamic vinegar and shows a lower total concentration of phenolic compounds compared to other samples of the same category. In the category of Balsamic Vinegar of Modena, samples No. 5 and No. 8 contained more than two times higher total concentrations of phenolic compounds than samples No. 6 and No. 7. The samples from the category of “premium” BVM show a good agreement of total concentrations that are even higher than in TBVM.

The highest total concentration of phenolic compounds was not observed in TBVM, but in “premium” BVM (75.6 ± 13.4 mg/L versus 56.7 mg/L, respectively), which can be attributed to the only one sample analyzed in the TBVM category in the study. There are, however, still some substances that are presented in significantly higher concentration in TBVM, than in any other sample analyzed. These compounds are 4-hydroxybenzaldehyde, syringaldehyde, 4-hydroxy-3-methoxycinnamaldehyde, and syringic acid. Therefore, these substances can be potential candidates for markers of the quality of balsamic vinegar samples.

## 4. Materials and Methods

### 4.1. Chemical and Reagents

Ethyl gallate, 4-hydroxybenzaldehyde, vanillin, tyrosol, protocatechuic aldehyde, syringaldehyde, pyrogallol, 4-hydroxy-3-methoxycinnamaldehyde, 4-methylcatechol, tryptophol, ethyl 3,4-dihydroxycinnamate, 2,6-dimethoxyphenol, ethyl vanillate, homovanillyl alcohol, eugenol, 4-hydroxy-3-methoxyphenylacetone, salicylaldehyde, coniferyl alcohol, epicatechin, catechin, scopoletin, *p*-coumaric acid, caffeic acid, resveratrol, syringic acid, rutin, gallic acid, salicylic acid, protocatechuic acid, 2-methoxy-4-vinylphenol, 4-vinylphenol, 4-ethylguaiacol were purchased from Sigma-Aldrich (Steinheim, Germany). All chemicals were used in the highest purity available. Acetonitrile and methanol (gradient grade for liquid chromatography) were also purchased from Sigma-Aldrich. Deionized water was produced by Mili-Q^®^ Reference System (Merck KGaA, Darmstadt, Germany). Sodium chloride was purchased by Lach-Ner (Neratovice, Czech Republic). Standard solution of *n*-alkanes C8-C33 was purchased by CPAchem (Stara Zagora, Bulgaria). Technical gases, i.e., helium 4.6, hydrogen, and nitrogen, were purchased from Linde Gas (Prague, Czech Republic).

### 4.2. Samples

Fourteen balsamic vinegar samples (Table 1) were purchased from Czech e-shops and local markets. The premium vinegars were not produced by traditional methods and therefore cannot be designated as TBVM but were rather produced by mixing aged wine vinegar with the must of cooked grapes (samples 9–13). The individual premium products differ mainly in the type of added vinegar and aging in barrels. For instance, sample 9 matured in French oak casks from 1900s and sample 13 matured in a series of antique small casks from the 1700s.

### 4.3. Gas Chromatography

#### 4.3.1. Selection of Extraction Technique

In order to concentrate VOCs prior to chromatographic analysis, the possibilities of static headspace extraction and solid-phase microextraction [41] were first tested, and the results from both extraction techniques were compared. The values of the total number of peaks and the total peak area in the chromatogram after separation were chosen as decision criteria. The extraction technique that better suited the requirements of the task was selected. 

##### Sample Preparation before Extraction

Three sample preparation options were tested prior to extraction: untreated sample, water diluted sample, and sample diluted with saturated salt solution (NaCl). The volume of sample dispensed into the headspace (HS) vial was 1 mL. The volume of distilled water or saturated NaCl solution was 4 mL. The HS vials were sealed with a Teflon septum.

##### Static Headspace Extraction

The incubation temperature was 95 °C, and the incubation time was chosen as 20 min. The volume of vapor phase collected after sample incubation for injection into the injection port of the gas chromatograph was 500 μL.

##### Headspace Solid-Phase Microextraction

Two extraction fibers were tested: 100 μm PDMS and 50/30 μm DVB/CAR/PDMS (Supelco, Bellefonte, PA, USA). The extraction fibers were heat-cleaned at 250 °C for 5 min immediately before extraction. The sample incubation time was 20 min. The extraction temperature was 50 °C and 95 °C, respectively, and the extraction time was 60 min. The extracted analytes were then desorbed in the injection port of the gas chromatograph at 230 °C for 15 s.

Based on a comparison of the results (see Results, Section 2.1) obtained by HS extraction and HS-SPME extraction, the HS-SPME method using 50/30 μm DVB/CAR/PDMS fiber was chosen for the extraction of VOCs from the vinegar samples. This extraction method was further optimized (see Section 4.3.2).

#### 4.3.2. Headspace Solid-Phase Microextraction Technique—Optimization

Vinegar sample No. 5 was selected for optimization of the headspace solid-phase microextraction method. The optimization was performed using the central composite design created in Statistica software (version 12, StatSoft CR, Prague, Czech Republic). Three parameters were optimized: extraction temperature (in the range 40–120 °C), extraction time (in the range 10–90 min), and volume of saturated NaCl solution added to the sample (in the range 0–9 mL). A total of 20 runs were performed at different extraction conditions (see Supplementary Table S3). Saturated NaCl solution was added to 1 mL of sample in a headspace vial sealed with a septum cap. Each sample was incubated at the extraction temperature for 20 min, after which the VOCs were extracted by DVB/CAR/PDMS fiber for the selected extraction time. The obtained extract was desorbed in the injection port of the gas chromatograph for 15 s. The results were evaluated by response surface method in Statistica software.

#### 4.3.3. Gas Chromatography Conditions

Separations of the obtained extracts were performed by gas chromatography. A GC2010 gas chromatograph (Shimadzu, Kyoto, Japan) equipped with a CombiPAL autosampler (CTC Analytics, AG, Zwingen, Switzerland) and an SLB-5MS capillary column (length 30 m, inner diameter 0.25 mm, film thickness 0.25 μm; Supelco, Bellefonte, PA, USA) was coupled to a QP 2010 Plus mass spectrometer (MS) and a flame ionization detector (FID), both from Shimadzu (Kyoto, Japan). Helium 4.6 was used as the carrier gas at a constant linear velocity of 30 cm·s^−1^. The split ratio was set to 1:5. The temperature of the injector was maintained at 230 °C. The temperature gradient was programmed as follows: the initial temperature was 40 °C (3 min) and then increased at 2 °C∙min^−1^ to 250 °C (5.5 min) with a total analysis time of 60 min.

FID conditions: The detector temperature was set to 270 °C.

MS conditions: The interface temperature and the ion source temperature were maintained both at 200 °C. The mass spectrometer was operated in electron ionization mode (70 eV), and the detection of ions was performed in full scan mode at a range of 33–450 *m*/*z*.

### 4.4. Liquid Chromatography

Stock solutions of the standards (1 mg/mL each) were prepared by weighing the appropriate amount of the compound and dissolving in methanol, which provided good solubility of the standards. Samples were diluted by deionized water (1:1, *v*/*v*) and centrifuged (10,000 rpm, 15 min) before HPLC analysis. HPLC analysis of phenolic acids was carried out using an Agilent Infinity II PRIME (Agilent, Palo Alto, CA, USA) system coupled with single quadrupole mass spectrometric detector Agilent iQ/MSD. All the other compounds were analysed using an Agilent 1200 Series (Agilent, Palo Alto, CA, USA) coupled with tandem mass spectrometric detector QTrap 4500 (Sciex, Framingham, MA, USA). A Luna C18 column (150 × 3.0 mm, 3 µm) (Phenomenex, Torrance, CA, USA) was used for all HPLC separation. The mobile phase was a mixture of acetonitrile and deionized water. The gradient for both methods was as follows: 3–48.4% B from 0 to 35 min, 48.4–100% B from 35 to 35.1 min, 100% B from 35.1 to 38 min, 100–3% B from 38 to 38.1 min, 3% B from 38.1 to 40 min. The injection volume was 3 µL. The flow rate was set to 0.5 mL/min. The temperature of the column was set to 40 °C. 

Phenolic acids were ionized using ESI source in negative mode. Detection by single quadrupole was performed in SIM mode. The following *m*/*z* were measured: gallic acid—169; protocatechuic acid—153; caffeic acid—179; *p*-coumaric acid—163; syringic acid—197; salicylic acid—137.

Other phenolic compounds were measured by MS/MS with ESI ionization. Optimization of MS/MS parameters was performed (see Results, Section 2.2). Detection was performed in negative mode using scheduled multiple-reaction monitoring (MRM) mode with time window 120 s.

The quantification of phenolic acids in the vinegar samples was performed by the standard addition method. Others phenolic compounds were quantified using the external calibration curve.

## 5. Conclusions

In this study, we identified and quantified 35 phenolic compounds in 14 different balsamic vinegar samples by the optimized HPLC method coupled with MS/MS and MS. The main phenolic compounds in balsamic vinegars are gallic acid, protocatechuic acid, caffeic acid, and *p*-coumaric acid. Significant differences in the total content of phenolic compounds were found between the types of balsamic vinegar. The HS-SPME-GC-MS method was used for identification and the HS-SPME-GC-FID method was used for the semi-quantitative analysis of 13 volatile phenolic compounds in balsamic vinegar samples. By comparing the results of the analysis achieved by both developed methods, i.e., GC-MS and HPLC-MS/MS, the comprehensive characterization of the samples was achieved, which can be used for the evaluation of the category of balsamic vinegars. Furthermore, several compounds (i.e., 4-hydroxybenzaldehyde, syringaldehyde, 4-hydroxy-3-methoxycinnamaldehyde, and syringic acid) were found to be potential candidates for markers of the quality of balsamic vinegar samples with respect to the content of the phenolic compounds. The profiles of phenolic compounds determined by gas and liquid chromatography could also be used to assess the authenticity of balsamic vinegar samples or to check for adulteration of these products, but this would first require the analysis of a larger pool of balsamic vinegars, in particular premium quality vinegars.

## Figures and Tables

**Table 1:** Balsamic vinegar samples (1–4 Greek balsamic vinegars, 5–8 balsamic vinegars of Modena, 9–13 “premium” balsamic vinegars of Modena, 14 traditional balsamic vinegar of Modena).

Sample	Trademark	Type	Approximate Price (EUR/100 mL)
1	Galaxy	White balsamic vinegar	0.7
2	Galaxy	Balsamic vinegar	0.6
3	Agia Triada	Balsamic vinegar (with thyme honey)	2.0
4	Agia Triada	Balsamic vinegar	1.5
5	San Fabio	BVM	0.3
6	Italiamo	BVM	0.9
7	Italiamo	BVM	0.8
8	Deluxe	BVM	1.4
9	Giuseppe Giusti	Silver medal BVM	11.0
10	Giuseppe Giusti	2 × Gold medals BVM	15.5
11	Giuseppe Giusti	3 × Gold medals BVM	23.0
12	Giuseppe Giusti	4 × Gold medals BVM	35.0
13	Giuseppe Giusti	5 × Gold medals BVM	53.0
14	Acetaia Casanova	TBVM	70.0

**Table 2:** Comparison of the content (mean; standard deviation; mg/L) of the major phenolic compounds from different studies. Our results are presented for each type of balsamic vinegar separately. Greek vinegars (*n* = 3; sample No. 1 was not included because a lack or different content of these compounds), BVM (*n* = 4), “premium” BVM (*n* = 5), TBVM (*n* = 1), [8] (*n* = 8), [16] (*n* = 6), [14] (*n* = 1). Average values from current study are calculated for samples containing the compounds in higher levels than limits of quantification (according to Table S6).

Compound	Greek Vinegars	BVM	“Premium” BVM	TBVM	Barnaba et al. [8]	Natera et al. [16]	Liu et al. [14]
Gallic acid	6.97 ± 4.03	11.61 ± 9.43	33.53 ± 9.33	21.42 ± 0.16	12.4 ± 11.8	64.50 ± 10.20	12.56 ± 0.86
Protocatechuic acid	1.69 ± 0.23	3.48 ± 1.00	12.06 ± 1.73	5.09 ± 1.42	6.11 ± 7.14	13.40 ± 29.94	3.29 ± 0.05
Caffeic acid	0.86 ± 0.43	1.52 ± 1.32	10.22 ± 3.21	1.61 ± 0.10	3.14 ± 3.17	28.70 ± 12.40	3.58 ± 0.14
*p*-Coumaric acid	0.47 ± 0.37	1.00 ± 1.02	7.09 ± 4.09	4.707 ± 0.69	2.45 ± 2.52	22.40 ± 33.00	1.97 ± 0.05
Salicylic acid	0.26 ± 0.01	0.63 ± 0.13	1.21 ± 0.45	1.51 ± 0.05	0.29 ± 0.26		
Syringic acid	n.d.	1.90 ± 1.81	2.71 ± 0.57	8.14 ± 1.04	1.05 ± 1.11	n.d.	
Pyrogallol	0.80 ± 0.03	0.80 ± 0.23	1.06 ± 0.26	1.12 ± 0.003			
Ethyl gallate	1.13 ± 0.41	0.96 ± 0.63	3.44 ± 1.47	2.63 ± 0.04			
Tyrosol	0.99 ± 0.19	1.88 ± 1.52	1.85 ± 1.16	2.906 ± 0.08	0.27 ± 0.36	109.40 ± 60.10	
Protocatechuic aldehyde	0.32 ± 0.08	0.92 ± 1.07	2.10 ± 1.44	4.62 ± 0.07	6.41 ± 8.35	15.70 ± 2.40	

n.d.—not detected.

## Data Availability

The data presented in this study are available in Supplementary Materials.

## Supplementary Materials

Table S1: Selection of extraction method—results of the analysis of sample No. 5 using static headspace extraction, Table S2: Selection of extraction method—results of the analysis of sample No. 5 using HS-SPME, Table S3: Central composite design used to optimize the extraction conditions of the HS-SPME method along with the corresponding observed responses, Table S4: Semi-quantitative content of phenolic compounds obtained by GC-FID analysis, Table S5: Optimization of MS/MS conditions for LC/MS/MS analysis of phenolic compounds, Table S6: The content of phenolic compounds in different samples of balsamic vinegars obtained by HPLC analysis, Table S7: Characteristics of calibration curves (slope and its relative standard deviation, RSD), linear ranges, coefficient of determination R^2^, and limits of quantification, LOQ.

## Sample Availability

Samples of the balsamic vinegars are available in limited amounts upon request from the authors.
