# Supplementary material for: Comparison of Phenolic Profile of Balsamic Vinegars Determined Using Liquid and Gas Chromatography Coupled with Mass Spectrometry
Source: Molecules. 2022 Feb 17;27(4):1356. doi: 10.3390/molecules27041356 (PMC8874619; doi:10.3390/molecules27041356)
Supplement: Supplementary file 1 [file molecules-27-01356-s001.zip › molecules-1589545-supplementary.pdf]

Electronic Supplementary Material

Comparison of Phenolic Profile of Balsamic Vinegars Determined using Liquid and Gas Chromatography Coupled with Mass Spectrometry

Michal Kašpar, Tomáš Bajer, Petra Bajerová, and Petr Česla

Department of Analytical Chemistry, Faculty of Chemical Technology, University of Pardubice, Studentská 573, CZ-53210 Pardubice, Czech Republic; michal.kaspar3@student.upce.cz (M.K.); tomas.bajer@upce.cz (T.B.); petra.bajerova@upce.cz (P.B.); petr.cesla@upce.cz (P.C.)

Table S1 Selection of extraction method - results of the analysis of sample No. 5 using static headspace extraction; NoP number of peaks in chromatogram; TPA total peak area in chromatogram.

Sample preparation	NoP	TPA
water diluted	38	84 864
diluted with saturated NaCl solution	22	89 463
untreated	31	188 746

Table S2 Selection of extraction method - results of the analysis of sample No. 5 using HS-SPME; NoP number of peaks in chromatogram; TPA total peak area in chromatogram.

Sample preparation	Extraction temperature [°C]	NoP		TPA	
		DVB/CAR/PDMS	PDMS	DVB/CAR/PDMS	PDMS
untreated	50	121	132	1 473 291	183 278
	95	81	102	449 938	257 742
water diluted	50	100	115	1 128 237	194 829
	95	130	49	235 246	70 112
diluted with saturated NaCl solution	50	112	124	1 175 967	358 769
	95	128	173	343 073	254 500

Table S3 Central composite design used to optimize the extraction conditions of the HS-SPME method along with the corresponding observed responses.

Run	Extraction conditions			Response
	T_{ext} [°C]	t_{ext} [min]	$V_{sat.\ NaCl}$ [mL]	NoP
1	40	10	1	77
2	40	10	9	91
3	40	90	1	95
4	40	90	9	114
5	100	10	1	127
6	100	10	9	90
7	100	90	1	131
8	100	90	9	134
9	40	50	5	104
10	100	50	5	125
11	70	10	5	103
12	70	90	5	125
13	70	50	1	110
14	70	50	9	120
15	70	50	5	118
16	70	50	5	112
17	70	50	5	119
18	70	50	5	113
19	120	40	0	49
20	120	40	3	34

T_{ext} = extraction temperature; t_{ext} = extraction time; $V_{sat.\ NaCl}$ = volume of saturated NaCl solution; NoP = Number of Peaks in the chromatogram

Table S4 Semi-quantitative content of phenolic compounds (average % of the peak area from the sum of all peak areas of volatile compounds; n=2) obtained by GC-FID analysis.

Compound	1	2	3	4	5	6	7	8	9	10	11	12	13	14
4-Ethylphenol					0.807 ± 0.052					0.968 ± 0.008				0.601 ± 0.063
Methyl salicylate			0.023 ± 0.002	0.017 ± 0.001	0.248 ± 0.013			0.592 ± 0.023		0.050 ± 0.010	0.054 ± 0.004	0.047 ± 0.001	0.061 ± 0.002	0.053 ± 0.001
Isopseudocumenol			0.060 ± 0.004											
4-Ethylguaiacol					0.389 ± 0.007	0.091 ± 0.003		0.036 ± 0.017	0.033 ± 0.001	0.255 ± 0.026	0.097 ± 0.018	0.023 ± 0.009	0.056 ± 0.002	0.138 ± 0.010
Thymol			2.410 ± 0.034	3.050 ± 0.069										
4-Vinylguaiacol								0.083 ± 0.002			0.055 ± 0.001	0.060 ± 0.001	0.044 ± 0.012	
1-Hydroxy-3,4,5-trimethylbenzene			0.052 ± 0.002											
Eugenol	0.016 ± 0.002		2.908 ± 0.107	5.689 ± 0.104	0.042 ± 0.011									0.082 ± 0.006
Allyl cresol isomer			0.066 ± 0.003	0.084 ± 0.003										
Vanilin	0.120 ± 0.031	0.144 ± 0.002												
trans-Isoeugenol				0.029 ± 0.001										
2,6-Di-*tert*-butyl-methylphenol										0.251 ± 0.052	0.170 ± 0.014		0.164 ± 0.030	
2,4-Di-*tert*-butylphenol					0.282 ± 0.011	0.068 ± 0.007	0.460 ± 0.049		0.183 ± 0.001	0.353 ± 0.014				

Table S5 Optimization of MS/MS conditions for LC/MS/MS analysis of phenolic compounds.

Compound	DP [V]	CE [V]	CXP [V]	MRM Transitions
Ethyl gallate	-95	-30	-13	197/124
4-Hydroxybenzaldehyde	-100	-34	-3	121/92
Vanillin	-55	-26	-11	151/92
Tyrosol	-60	-22	-9	137/106
Protocatechuic aldehyde	-85	-30	-7	137/108
Syringaldehyde	-65	-28	-13	181/151
4-Hydroxy-3-methoxycinnamaldehyde	-75	-26	-5	177/133
Pyrogallol	-75	-26	-11	125/79
4-Methylcatechol	-80	-26	-11	123/105
Tryptophol	-70	-22	-13	160/130
Ethyl 3,4-dihydroxycinnamate	-65	-28	-11	207/135
2,6-Dimethoxyphenol	-105	-6	-1	154/122
Ethyl vanillate	-60	-20	-5	195/180
Homovanillyl alcohol	-65	-38	-7	167/121
Eugenol	-100	-24	-11	164/121
4-Hydroxy-3-methoxyphenylacetone	-70	-30	-17	179/121
Salicylaldehyde	-100	-30	-7	122/93
Coniferyl alcohol	-55	-18	-13	179/146
Epicatechin	-80	-22	-9	289/245
Catechin	-110	-20	-9	289/245
Scopoletin	-75	-34	-5	191/104
Resveratrol	-100	-28	-5	227/185
Rutin	-175	-52	-15	609/300
2-Methoxy-4-vinylphenol	-45	-18	-13	149/134
4-Vinylphenol	-60	-32	-9	119/93
4-Ethylguaiacol	-50	-28	-11	151/121

Table S6 The content of phenolic compounds (mg/L; mean ± standard deviation; n=3) in different samples of balsamic vinegars obtained by HPLC analysis.

Compound	1	2	3	4	5	6	7	8	9	10	11	12	13	14
Ethyl gallate	0.052 ± 0.010	1.598 ± 0.025	0.819 ± 0.010	0.967 ± 0.012	1.049 ± 0.013	0.934 ± 0.024	0.152 ± 0.004	1.689 ± 0.01	1.009 ± 0.017	4.062 ± 0.115	4.831 ± 0.068	4.022 ± 0.044	3.253 ± 0.026	2.630 ± 0.040
4-Hydroxybenzaldehyde	0.016 ± 0.001	0.068 ± 0.002	0.101 ± 0.001	0.080 ± 0.001	0.066 ± 0.004	0.065 ± 0.005	0.095 ± 0.002	0.319 ± 0.004	0.034 ± 0.001	0.109 ± 0.010	0.206 ± 0.004	0.207 ± 0.006	0.296 ± 0.005	0.489 ± 0.012
Vanillin	3.132 ± 0.094	2.870 ± 0.017	0.020 ± 0.001	0.012 ± 0.001	0.027 ± 0.001	0.029 ± 0.001	0.016 ± 0.001	0.055 ± 0.002	0.008 ± 0.001	0.029 ± 0.004	0.023 ± 0.001	0.040 ± 0.001	0.099 ± 0.001	0.306 ± 0.011
Tyrosol	1.115 ± 0.028	0.923 ± 0.018	0.844 ± 0.049	1.212 ± 0.044	3.708 ± 0.104	2.463 ± 0.054	0.262 ± 0.010	1.100 ± 0.030	1.133 ± 0.025	3.742 ± 0.541	2.104 ± 0.022	0.741 ± 0.017	1.525 ± 0.027	2.906 ± 0.081
Protocatechuic aldehyde	0.055 ± 0.001	0.229 ± 0.005	0.393 ± 0.011	0.348 ± 0.011	0.422 ± 0.018	0.155 ± 0.003	0.593 ± 0.013	2.506 ± 0.034	0.396 ± 0.008	0.886 ± 0.028	2.263 ± 0.025	3.408 ± 0.047	3.567 ± 0.060	4.622 ± 0.072
Syringaldehyde	*5.000 ± 0.200	0.258 ± 0.011	0.024 ± 0.001	0.018 ± 0.001	0.035 ± 0.001	0.023 ± 0.001	0.019 ± 0.001	0.064 ± 0.001	0.014 ± 0.001	0.058 ± 0.007	0.043 ± 0.001	0.054 ± 0.002	0.181 ± 0.011	0.656 ± 0.012
4-Hydroxy-3-methoxycinnamaldehyde			0.045 ± 0.001	0.054 ± 0.005	0.044 ± 0.003	0.081 ± 0.004	0.049 ± 0.002	0.106 ± 0.007	0.048 ± 0.004	0.084 ± 0.005	0.082 ± 0.011	0.119 ± 0.005	0.158 ± 0.008	0.532 ± 0.010
Pyrogallol	0.101 ± 0.012	0.786 ± 0.007	0.830 ± 0.008	0.773 ± 0.004	0.706 ± 0.001		0.622 ± 0.004	1.061 ± 0.014	0.596 ± 0.001	1.239 ± 0.024	1.182 ± 0.004	1.144 ± 0.002	1.129 ± 0.012	1.118 ± 0.003
4-Methylcatechol	*8.000 ± 0.100	0.025 ± 0.004	0.438 ± 0.012	0.514 ± 0.015	0.416 ± 0.007	0.222 ± 0.005	0.300 ± 0.001	0.167 ± 0.014	0.157 ± 0.008	0.025 ± 0.001	0.042 ± 0.001	0.052 ± 0.001	0.053 ± 0.001	0.046 ± 0.001
Tryptophol		*1.000 ± 0.100	<LOQ	<LOQ										
Ethyl 3,4-dihydroxycinnamate	0.008 ± 0.001	0.083 ± 0.003	0.040 ± 0.001	0.064 ± 0.001	0.748 ± 0.002	0.087 ± 0.003	0.009 ± 0.001	0.039 ± 0.001	0.023 ± 0.001	0.685 ± 0.013	0.751 ± 0.007	0.135 ± 0.002	0.374 ± 0.003	0.108 ± 0.002
2,6-Dimethoxyphenol		*12.000 ± 0.200	<LOQ	<LOQ	0.156 ± 0.005	0.007 ± 0.001	<LOQ	<LOQ	<LOQ	0.132 ± 0.013	0.124 ± 0.002	0.017 ± 0.003	0.020 ± 0.003	0.035 ± 0.002
Ethyl vanillate	*0.297 ± 0.072	*3.495 ± 0.057	*1.103 ± 0.036	*1.759 ± 0.039	*34.159 ± 0.914	*4.577 ± 0.285	*0.170 ± 0.039	*3.718 ± 0.117	*1.448 ± 0.069	*25.911 ± 5.234	*19.680 ± 0.950	*5.737 ± 0.167	*14.625 ± 0.252	*14.816 ± 0.165
Homovanillyl alcohol	<LOQ	<LOQ	0.073 ± 0.008	0.021 ± 0.003	0.105 ± 0.007	0.067 ± 0.001	0.003 ± 0.0001	0.079 ± 0.008	<LOQ	0.161 ± 0.016	0.079 ± 0.008	0.091 ± 0.003	0.088 ± 0.008	0.080 ± 0.004
Eugenol	0.076 ± 0.004	0.056 ± 0.008	0.086 ± 0.007	0.063 ± 0.004	0.324 ± 0.015	0.087 ± 0.007	0.109 ± 0.001	0.197 ± 0.052	0.194 ± 0.027	0.189 ± 0.041	0.226 ± 0.040	0.060 ± 0.007	<LOQ	<LOQ
4-Hydroxy-3-methoxyphenylacetone	*4.000 ± 0.300	*4.000 ± 0.200			*3.000 ± 0.300	0.045 ± 0.001		0.011 ± 0.001	<LOQ	<LOQ	<LOQ			0.006 ± 0.001
Salicylaldehyde					<LOQ					<LOQ	<LOQ			
Epicatechin	*2.230 ± 0.307	*2.305 ± 0.150	<LOQ						*2.310 ± 0.150		<LOQ		<LOQ	
Catechin	*2.110 ± 0.410	*1.790 ± 0.160	<LOQ			<LOQ								

Scopoletin		*43.130 ± 1.350	*7.050 ± 0.110	*3.390 ± 0.050	*4.250 ± 0.070	*10.130 ± 0.140	*2.250 ± 0.010	<LOQ		*6.406 ± 0.290	*5.830 ± 0.070	*5.290 ± 0.050	*9.620 ± 0.100	*36.930 ± 0.910
p-Coumaric acid		0.366 ± 0.035	0.883 ± 0.051	0.167 ± 0.002	2.466 ± 0.016	0.482 ± 0.019	0.869 ± 0.057	7.185 ± 0.054	5.797 ± 0.556	4.096 ± 0.071	14.05 ± 0.152	4.268 ± 0.062	7.222 ± 0.097	4.707 ± 0.686
Caffeic acid		1.102 ± 0.04	1.110 ± 0.047	0.360 ± 0.006	3.325 ± 0.072	0.764 ± 0.018	1.637 ± 0.118	6.108 ± 0.038	5.772 ± 0.002	7.85 ± 0.425	12.37 ± 0.503	13.03 ± 0.255	12.07 ± 0.412	1.607 ± 0.104
Resveratrol	*0.609 ± 0.110	*0.940 ± 0.132	*12.430 ± 0.250		<LOQ									
Syringic acid				<LOQ	1.399 ± 0.133	<LOQ	0.397 ± 0.001	3.915 ± 0.510		<LOQ	2.852 ± 0.472	2.079 ± 0.139	3.203 ± 0.151	8.140 ± 1.042
Rutin	*67.800 ± 10.960	*6.909 ± 0.852	*5.867 ± 0.155	*14.780 ± 0.836	*2.052 ± 0.186	*3.919 ± 0.299	*2.303 ± 0.363	*2.168 ± 0.060	*1.083 ± 0.161	*3.056 ± 0.556	*1.907 ± 0.139			
Gallic acid		10.160 ± 0.160	8.301 ± 0.117	2.449 ± 0.159	24.650 ± 0.380	7.985 ± 0.253	11.350 ± 1.160	22.770 ± 1.710	46.970 ± 1.390	24.060 ± 0.040	38.990 ± 2.500	27.370 ± 0.080	30.280 ± 1.420	21.420 ± 0.160
Salicylic acid			0.254 ± 0.059	0.271 ± 0.067	0.543 ± 0.116		<LOQ	0.725 ± 0.056	0.614 ± 0.025	0.857 ± 0.058	1.646 ± 0.002	1.388 ± 0.011	1.562 ± 0.098	1.515 ± 0.052
Protocatechuic acid			1.845 ± 0.113	1.526 ± 0.090	4.188 ± 0.130	<LOQ	2.777 ± 0.043		10.020 ± 0.470	10.020 ± 0.470	13.780 ± 2.260	13.800 ± 0.900	12.040 ± 0.390	5.090 ± 1.420
4-Vinylphenol		0.017 ± 0.003	0.031 ± 0.002	0.012 ± 0.002	0.034 ± 0.002	<LOQ		0.053 ± 0.001	0.027 ± 0.005	<LOQ	0.214 ± 0.014	0.044 ± 0.007	0.044 ± 0.004	<LOQ

<LOQ – values are under Limit of Quantification according to **Table S7**; * - values given in μg/L

Table S7 Characteristics of calibration curves (slope and its relative standard deviation, RSD), linear ranges, coefficient of determination R^2, and limits of quantification, LOQ.

Compound	Slope	RSD [%]	Linear range [mg/L]	R^2	LOQ [µg/L]
Ethyl gallate	1673907.2	1.71	0.005 – 0.5	0.9962	5
4-hydroxybenzaldehyde	57802577.3	0.47	0.001 – 0.5	0.9997	1
Vanillin	11981151.5	0.36	0.001 – 1	0.9998	1
Tyrosol	610790.6	0.64	0.001 – 1	0.9992	1
Protocatechuic aldehyde	5749011.3	1.16	0.005 – 1	0.9979	5
Syringaldehyde	7677901.1	0.68	0.001 – 0.5	0.9993	1
4-Hydroxy-3-methoxycinnamaldehyde	294700.9	2.01	0.05 – 1	0.9960	40
Pyrogallol	806299.3	1.32	0.5 – 10	0.9988	50
4-Methylcatechol	469121.4	1.62	0.005 – 0.5	0.9966	5
Tryptophol	1812422.5	0.62	0.001 – 0.5	0.9994	1
Ethyl 3,4-dihydroxycinnamate	31669129.1	0.60	0.001 – 0.5	0.9994	1
2,6-Dimethoxyphenol	287266.8	1.18	0.005 – 0.5	0.9982	5
Ethyl vanillate	19885553.2	0.97	0.0001 – 1	0.9979	0.1
Homovanillyl alcohol	81047.9	2.10	0.05 – 1	0.9956	50
Eugenol	24693.3	3.07	0.05 – 0.5	0.9953	50
4-Hydroxy-3-methoxyphenylacetone	922518.6	0.87	0.005 – 1	0.9988	2
Salicylaldehyde	51596.1	0.81	0.1 – 10	0.9993	500
Coniferyl alcohol	602723.3	1.54	0.005 – 0.5	0.9969	5
Epicatechin	2386182.3	0.34	0.005 – 1	0.9998	2
Catechin	1712184.0	0.27	0.005 – 1	0.9999	1
Scopoletin	7180463.9	0.55	0.001 – 1	0.9994	1
Resveratrol	6316269.9	1.31	0.001 – 1	0.9968	0.5
Rutin	1497812.4	0.31	0.001 – 0.5	0.9998	1
2-Methoxy-4-vinylphenol	82873.5	3.12	0.05 – 1	0.9903	50
4-Vinylphenol	139707.8	1.18	0.01 – 1	0.9982	10
4-Ethylguaiacol	3475.2	1.44	0.5 – 10	0.9985	500
